# Association of red blood cell distribution width–coefficient of variation with cranial ultrasound abnormalities in neonatal hyperbilirubinemia: a retrospective cross-sectional study

**DOI:** 10.3389/fped.2024.1488731

**Published:** 2025-01-29

**Authors:** Hongjuan Wei, Xin Chang, Jin Wang

**Affiliations:** ^1^ Department of Pediatric, Nanjing Lishui People's Hospital, Zhongda Hospital Lishui Branch, Southeast University, Nanjing, China; ^2^ Department of Ultrasound Medicine, Nanjing Lishui People's Hospital, Zhongda Hospital Lishui Branch, Southeast University, Nanjing, China

**Keywords:** cranial, red blood cell distribution width, neonatal hyperbilirubinemia, ultrasonography, neuroimaging

## Abstract

**Background:**

Neonatal hyperbilirubinemia frequently leads to severe neurological damage. Although cranial ultrasound (CUS) is crucial for assessing neonatal brain injury, the association between red blood cell distribution width-coefficient of variation (RDW-CV), a marker of red blood cell size variability, and cranial ultrasound abnormalities (CUAs) remains unclear.

**Objective:**

The primary aim of this study was to explore the impact of RDW-CV on CUAs in neonatal hyperbilirubinemia and to elucidate the potential clinical implications of this relationship.

**Methods:**

This retrospective cross-sectional study included 503 cases of neonatal hyperbilirubinemia at gestational age ≥35 weeks with available RDW-CV and CUS screening data at Nanjing Lishui People's Hospital. Multivariate logistic regression analysis and smooth curve fitting were used to estimate the association between RDW-CV and the risk of CUAs in neonatal hyperbilirubinemia.

**Results:**

This study found that the overall prevalence of CUAs in ultrasound images was 26.0%. Multivariate logistic regression analysis adjusted for risk factors revealed that a one-percent increase in RDW-CV increased the risk of CUAs by 23.0%. After conducting a sensitivity analysis of the three RDW-CV quantiles, the findings remained robust and consistent.

**Conclusions:**

The study concluded that a higher RDW-CV was associated with a proportional increase in the risk of CUAs. These results demonstrate the importance of RDW-CV in neonatal hyperbilirubinemia. Clinicians should consider this association when managing patients with high RDW-CV.

## Introduction

Jaundice is a common condition in newborn infants, affecting more than 80% of them (1). High bilirubin levels can lead to acute bilirubin encephalopathy and kernicterus, which are serious neurological complications. Therefore, careful monitoring of the cranial status in patients with neonatal hyperbilirubinemia is essential to prevent and manage these potential complications (2). CUS is an easily accessible and well-accepted first-line imaging modality that is widely used to screen preterm and high-risk infants for brain injury during the neonatal period (3). Various studies have sought to identify prognostic factors associated with CUAs. Preterm, intracranial infection, hypoxia and other factors have been shown to have independent predictive value (4). One potential predictive clinical variable is RDW-CV. RDW-CV is a vital blood parameter that serves as a key indicator of physiological health and plays a crucial role in assessing overall well-being and bodily function. Recently RDW-CV has been found to have prognostic value in subarachnoid hemorrhage and other intracranial diseases (5). In these studies, it was shown that increased value of RDW was an independent prognostic factor for poor outcome in these patients. We hypothesize that the incorporation of RDW will improve prediction of overall CUAs in neonatal hyperbilirubinemia. Although CUS screening is routinely performed for neonatal hyperbilirubinemia, the relationship between RDW-CV and CUAs in neonates remains unclear. Understanding the relationship between these factors is essential for improving medical interventions and treatment of neonates. Therefore, this study aimed to explore the potential correlation between neonatal RDW-CV and CUAs, with the goal of enhancing the care and management of neonatal health.

## Materials and methods

### Study population

**Figure 1:**
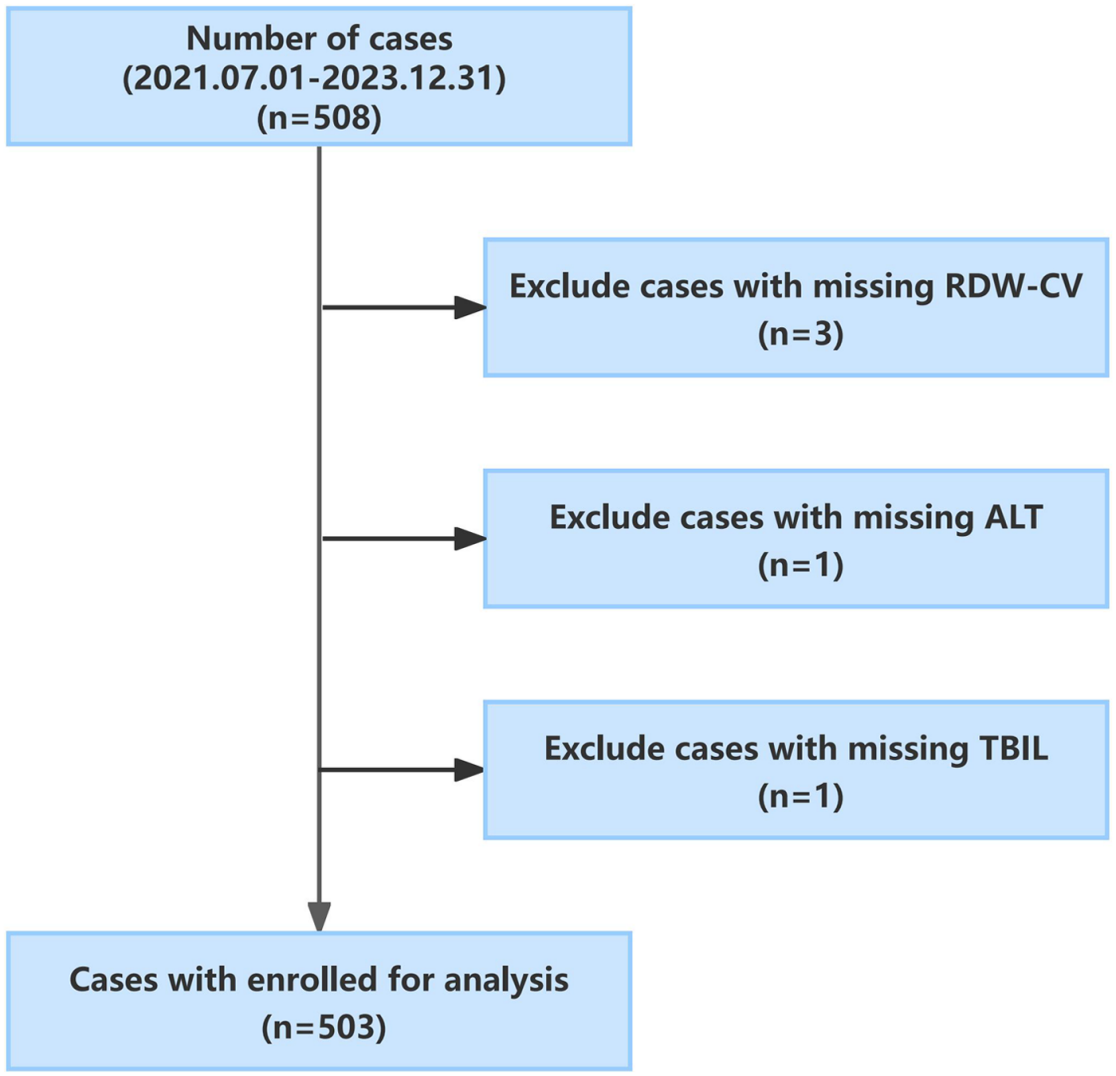
Flowchart for the study population.

This study adhered to the Strengthening the Reporting of Observational Studies in Epidemiology (STROBE) statement. This retrospective cross-sectional study included 508 patients with neonatal hyperbilirubinemia requiring phototherapy, who were admitted to the NICU of Nanjing Lishui People's Hospital (NJLSPH) between July 1, 2021, and December 31, 2023, with a gestational age of ≥35 weeks. Neonatal records were obtained from the hospital and retrieved from the electronic medical record system. The exclusion criteria were missing RDW-CV, alanine aminotransferase (ALT), and total bilirubin (TBIL) values. Ultimately, 503 patients were analyzed (Figure 1). This study was approved by the Research Ethics Committee of NJLSPH (Approval No 2024KY0726-01). As this study had a retrospective design and only de-identified and anonymized participant information was used, the need for written informed consent was waived by NJLSPH. This study was registered at the Chinese Clinical Trial Registry Center (Registration Number: ChiCTR 2400087550).

### Laboratory, ultrasound data collection and measures

The data of neonatal hyperbilirubinemia patients admitted to the NJLSPH between July 1, 2021, and December 31, 2023, were collected. The database contains information on demographics, laboratory values, and ultrasound findings. Neonatal demographics included sex, weeks of gestation, birth weight, weight at admission, age, blood type, parity, number of births, and mode of delivery. Laboratory values included hsCRP, RBC, HGB, HCT, MCV, MCH, RDW-CV, WBC, NEUT, LYMPH, MONO, PLT, PCT, MPV, TBIL, ALT, GGT, ALP, ALB, GLO, and A/G levels.

All data were tested in the biochemical laboratory of NJLSPH. Clinical chemistry and biochemical laboratory parameters (TBIL, ALT, GGT, ALP, ALB, GLO, and A/G) were measured using a biochemical analyzer (Beckman Coulter Trading Co. Ltd., China; Model: AU5800). Hematological variables (hsCRP, RBC, HGB, HCT, MCV, MCH, RDW-CV, and WBC) were measured using an instrument (BC-7500; Mindray Corporation, Shenzhen, China).

In accordance with the routine scheduling for CUS screenings, which are conducted on Tuesdays and Fridays, neonates admitted to the NICU underwent one or more ultrasound examinations during their hospital stay. Notably, the median age for these CUS examinations was on the second day of hospitalization. Ultrasonographic findings were categorized into two groups: normal and abnormal. CUAs included subependymal cysts, choroidal cysts, ventricular enlargement, periventricular-intraventricular hemorrhage (PVH-IVH), cerebellar hemorrhage, and cephalohematoma. PVH-IVH and cerebellar hemorrhage were classified as intracranial hemorrhage.

### Statistical analysis

All statistical analyses were conducted using R Statistical Software (Version 4.2.2, http://www.R-project.org, The R Foundation) and the Free Statistics Analysis Platform (Version 1.9, Beijing, China, http://www.clinicalscientists.cn/freestatistics). Statistical significance was defined as a *P*-value <0.05. Histogram distribution and Kolmogorov-Smirnov tests were used to assess the normality of the variables. Normally distributed continuous variables are reported as mean ± standard deviation, while skewed continuous variables are presented as median with interquartile range (IQR). Categorical variables are presented as frequencies (%). Statistical comparisons of continuous variables between groups were conducted using the independent samples Student's *t*-test or Mann-Whitney *U*-test, depending on the normality of the distribution. Categorical data were analyzed using the chi-square test or Fisher's exact test, as appropriate. Logistic regression analysis was used to investigate the relationship between RDW-CV and CUAs. RDW-CV was analyzed as a continuous variable and in tertiles. Confounding variables were selected based on expert judgment, previous scientific literature, or established associations with the outcomes of interest, with a minimum impact of 10% on the effect estimate. A series of models were constructed to control for potential confounders. Model I was adjusted for age and sex. Model II included additional adjustments for birth weight, gestational age, and delivery mode. In model III (primary model), further adjustments were made for TBIL, WBC, HCT, PLT, and ALT levels. Trends were tested using multivariate regression models, in which the median value of each tertile of RDW-CV was included as a continuous variable. A restricted cubic spline model was employed to generate smooth curves in order to investigate potential dose-response relationships between RDW-CV and CUAs. In this model, RDW-CV was treated as a continuous variable with four knots (5th, 35th, 65th, and 95th percentiles), as recommended by Harrell.

## Results

### Baseline characteristics of the study population

A total of 503 patients with a median age of 108.5 h (81.0, 155.7) were included in the analysis. The overall prevalence of CUAs was 26.04%. The baseline clinical characteristics of the patients included in the study are shown in Table 1.

**Table 1:** Baseline characteristics of the study population.

	Total (*n* = 503)	Non-CUAs (*n* = 372)	CUAs (*n* = 131)	*P*	Statistic
Sex, *n* (%)				0.428	0.628
Male	276 (54.9)	208 (55.9)	68 (51.9)		
Female	227 (45.1)	164 (44.1)	63 (48.1)		
Week of gestation, week	38.9 ± 1.3	39.0 ± 1.2	38.7 ± 1.4	0.013	6.148
Birth Weight, g	3,360.4 ± 426.9	3,348.5 ± 426.5	3,394.2 ± 427.8	0.293	1.109
Admission weight, g	3,199.3 ± 439.4	3,192.1 ± 437.9	3,219.7 ± 444.6	0.537	0.381
Age, h	108.5 (81.0, 155.7)	108.6 (81.9, 156.1)	107.4 (78.4, 147.5)	0.569	0.325
Diagnose, *n* (%)				0.322	0.980
Hyperbilirubinemia	420 (83.5)	307 (82.5)	113 (86.3)		
ABO hemolytic jaundice	83 (16.5)	65 (17.5)	18 (13.7)		
Blood type, *n* (%)				0.027	Fisher
O+	140 (28.0)	112 (30.4)	28 (21.4)		
A+	175 (35.0)	131 (35.5)	44 (33.6)		
B+	151 (30.2)	98 (26.6)	53 (40.5)		
AB+	33 (6.6)	27 (7.3)	6 (4.6)		
O-	1 (0.2)	1 (0.3)	0 (0)		
Parity, *n* (%)				0.957	Fisher
1	228 (45.3)	167 (44.9)	61 (46.6)		
2	125 (24.9)	94 (25.3)	31 (23.7)		
3	76 (15.1)	55 (14.8)	21 (16)		
4	40 (8.0)	31 (8.3)	9 (6.9)		
5	19 (3.8)	15 (4.0)	4 (3.1)		
6 + ^a^	15 (3.0)	10 (2.7)	5 (3.8)		
Number of births, *n* (%)				0.822	Fisher
1	284 (56.5)	212 (57.0)	72 (55.0)		
2	180 (35.8)	130 (34.9)	50 (38.2)		
3	36 (7.2)	28 (7.5)	8 (6.1)		
4	3 (0.6)	2 (0.5)	1 (0.8)		
Delivery mode, *n* (%)				0.644	Fisher
Vaginal delivery	322 (64.0)	237 (63.7)	85 (64.9)		
C—section	173 (34.4)	130 (34.9)	43 (32.8)		
Vaginal delivery to C—section	8 (1.6)	5 (1.3)	3 (2.3)		
hsCRP, mg/L	1.3 (0.8, 3.3)	1.3 (0.8, 3.1)	1.4 (0.8, 3.7)	0.718	0.131
RBC, 10e12/L	4.9 ± 0.6	4.9 ± 0.6	4.9 ± 0.6	0.467	0.529
HGB, g/L	171.8 ± 20.1	172.2 ± 20.1	170.7 ± 20.3	0.466	0.532
HCT,%	48.7 ± 6.0	48.9 ± 6.1	48.3 ± 5.8	0.332	0.942
MCV, fL	99.7 ± 4.7	99.7 ± 4.7	99.5 ± 5.0	0.573	0.318
MCH, pg	35.2 ± 2.0	35.2 ± 2.0	35.2 ± 1.9	0.954	0.003
RDW-CV,%	15.3 ± 1.1	15.2 ± 1.0	15.4 ± 1.3	0.014	6.035
WBC, 10^9^/L	10.6 ± 3.3	10.8 ± 3.4	10.1 ± 2.7	0.061	3.528
NEUT, 10^9^/L	4.2 (3.2, 5.8)	4.2 (3.2, 5.9)	4.2 (3.2, 5.4)	0.357	0.850
LYMPH, 10^9^/L	4.2 ± 1.4	4.2 ± 1.3	4.1 ± 1.4	0.617	0.251
MONO, 10^9^/L	1.1 ± 0.4	1.1 ± 0.4	1.0 ± 0.4	0.038	4.350
PLT, 10^9^/L	280.8 ± 81.2	283.6 ± 84.4	272.8 ± 71.1	0.190	1.721
PCT,%	0.3 ± 0.1	0.3 ± 0.1	0.3 ± 0.1	0.334	0.937
MPV, fL	10.1 ± 1.0	10.1 ± 1.0	10.2 ± 0.9	0.683	0.167
TBIL, umol/L	306.4 ± 52.7	307.6 ± 52.5	302.9 ± 53.3	0.378	0.780
ALT, U/L	16.3 ± 6.0	16.4 ± 6.0	16.1 ± 6.1	0.639	0.220
GGT, U/L	154.7 ± 68.0	156.9 ± 67.8	148.6 ± 68.5	0.231	1.437
ALP, U/L	161.8 ± 58.8	162.5 ± 60.9	159.6 ± 52.8	0.618	0.249
ALB, g/L	36.4 ± 3.1	36.4 ± 3.1	36.3 ± 2.9	0.773	0.083
GLO, g/L	22.6 ± 4.1	22.6 ± 4.3	22.6 ± 3.8	0.946	0.005
A/G	1.7 ± 0.4	1.7 ± 0.4	1.7 ± 0.4	0.683	0.166

Note: ^a^6+: Including pregnancy 6 times and above.

Table 2 presents the results of the multivariate logistic regression analysis examining the association between RDW-CV and CUAs. A higher RDW-CV was significantly correlated with an increased risk of CUAs. In the crude model, each 1% increase in RDW-CV was associated with a 25% increase in the risk of CUAs. After adjusting for all potential confounders, the risk of CUAs was significantly associated with RDW-CV (OR, 1.23; 95%CI: 1.01–1.48).

**Table 2:** Logistic regression analysis of RDW-CV and the risk of CUAs in neonatal hyperbilirubinemia.

	*n*. total	*N* (%)	Crude Model OR(95% CI)	*P*-value	Model Ⅰ OR(95% CI)	*P*-value	Model Ⅱ OR(95% CI)	*P*-value	Model Ⅲ OR(95% CI)	adj. *P* value
RDW-CV	503.0	131 (26)	1.25 (1.04–1.49)	0.016	1.24 (1.03–1.49)	0.021	1.22 (1.01–1.46)	0.041	1.23 (1.01–1.48)	0.037
Q1 13.4%≤RDW-CV<14.7%	152.0	33 (21.7)	1(Ref)		1(Ref)		1(Ref)		1(Ref)	
Q2 14.7%≤RDW-CV<15.5%	176.0	41 (23.3)	1.10 (0.65–1.84)	0.732	1.09 (0.64–1.83)	0.762	1.06 (0.62–1.82)	0.824	1.04 (0.60–1.79)	0.888
Q3 15.5%≤RDW-CV<20.8%	175.0	57 (32.6)	1.74 (1.06–2.87)	0.029	1.73 (1.05–2.87)	0.033	1.64 (0.97–2.75)	0.063	1.66 (0.98–2.81)	0.058
*P* for trend				0.024		0.026		0.050		0.045

Note: Data are presented as OR and 95% confidence interval (CI). In the crude model, no covariates were adjusted. Model I: Adjusted for age and sex. Model II: Adjusted for model I and for birth weight, week of gestation, and delivery mode. Model III: Adjusted for model II and for WBC, HCT, PLT, ALT, and TBIL.

**Figure 2:**
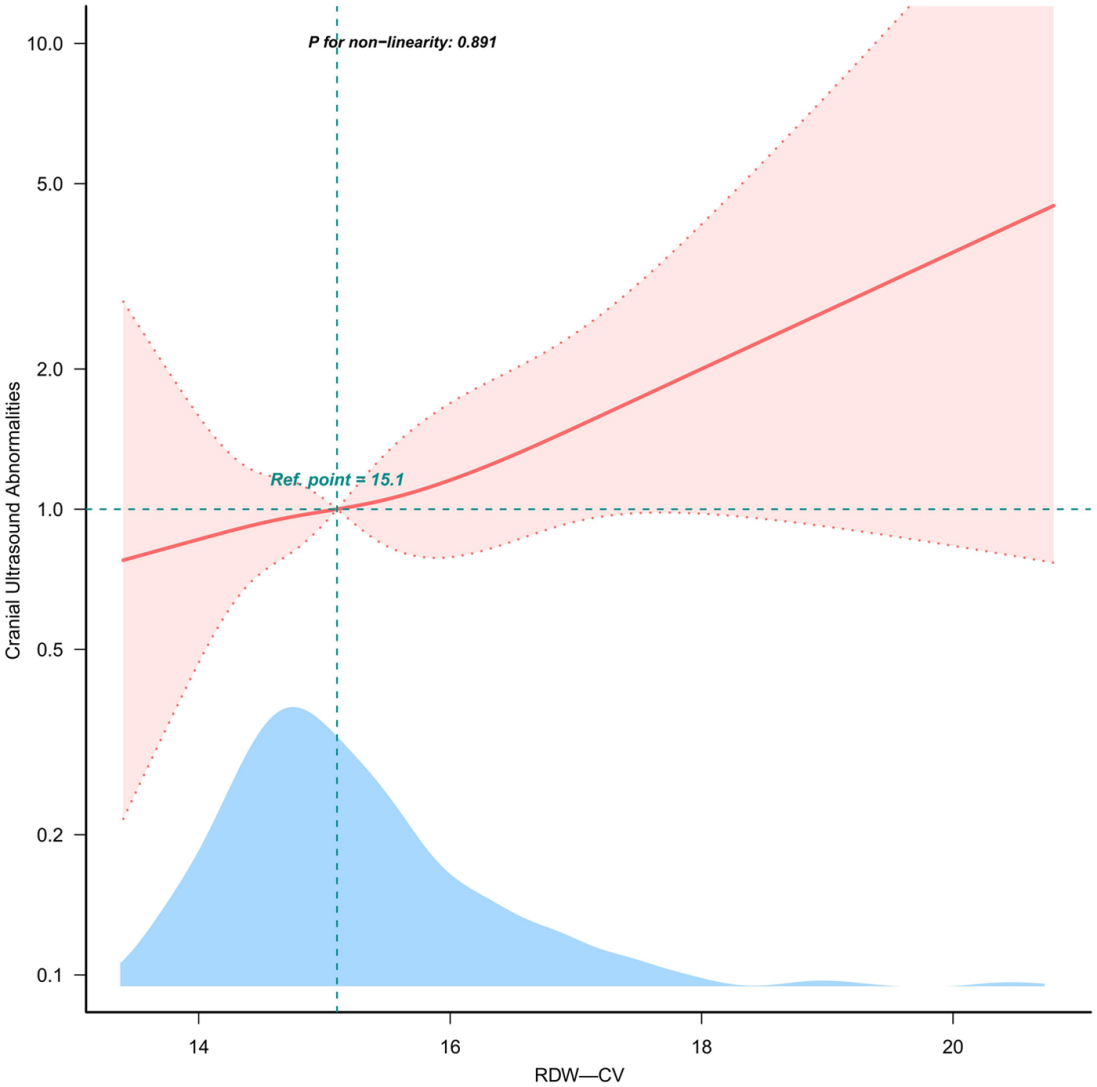
Linear dose-response relationship between RDW-CV and OR of CUAs. Note: Figure 2 presents restricted cubic spline plots illustrating the relationship between RDW-CV and CUAs outcomes after covariate adjustment. Background histograms (light blue) show the percentage of RDW-CV density distribution in the study population. The solid central lines represent the estimated adjusted odds ratios accompanied by shaded ribbons indicating 95% confidence intervals (CIs). The horizontal dashed lines represent the odds ratio of 1.0 (reference point). The reference point was the median RDW-CV (15.1%).

Evidence derived from the estimated dose-response curve demonstrated a significant linear relationship between RDW-CV and the risk of CUAs (Figure 2; *P* for nonlinearity = 0.891) based on restricted cubic spline analyses adjusted for multiple variables. With increasing RDW-CV levels, the risk of CUAs increased.

The E-value was calculated to evaluate the robustness of the findings against potentially unmeasured confounding factors. Our results were deemed statistically significant unless there was an unmeasured confounding variable, with the risk ratio for CUAs exceeding 1.452 (E-value). When RDW-CV was analyzed as a tertile variable, significant differences were observed between RDW-CV and the risk of CUAs after adjusting for all potential confounders (*P* for trend = 0.045). Compared with RDW-CV Q1 (13.4%≤RDW-CV<14.7%), the adjusted ORs for the risk of CUAs in Q2 (14.7%≤RDW-CV<15.5%) and Q3 (15.5%≤RDW-CV<20.8%) were 1.04 (95%CI: 0.60–1.79) and 1.66 (95%CI: 0.98–2.81), respectively.

## Discussion

This retrospective cross-sectional study, conducted in NJLSPH, included cases of neonatal hyperbilirubinemia at ≥35 weeks. This study found a significant association between RDW-CV and CUAs in neonatal hyperbilirubinemia (Table 2).

RDW is a hematologic parameter routinely measured in clinical blood tests, and it reflects the volume heterogeneity of peripheral red blood cells (RBCs) (6). Compared with observing RBCs on a blood smear with the naked eye, it can more objectively reflect the degree of the unequal size of red blood cells (7). Variations in RDW are attributed to multiple factors, including nutritional deficiency (vitamin B12), bone marrow suppression, hemolysis, and splenic sequestration (8). RDW is associated with various diseases (9, 10), such as cardiovascular disease, diabetes, and kidney disease. As a clinical biomarker, RDW plays an increasing role in predicting outcomes in hematologic malignancies, as well as lung, breast, and gastrointestinal cancer (11–13).

**Figure 3:**
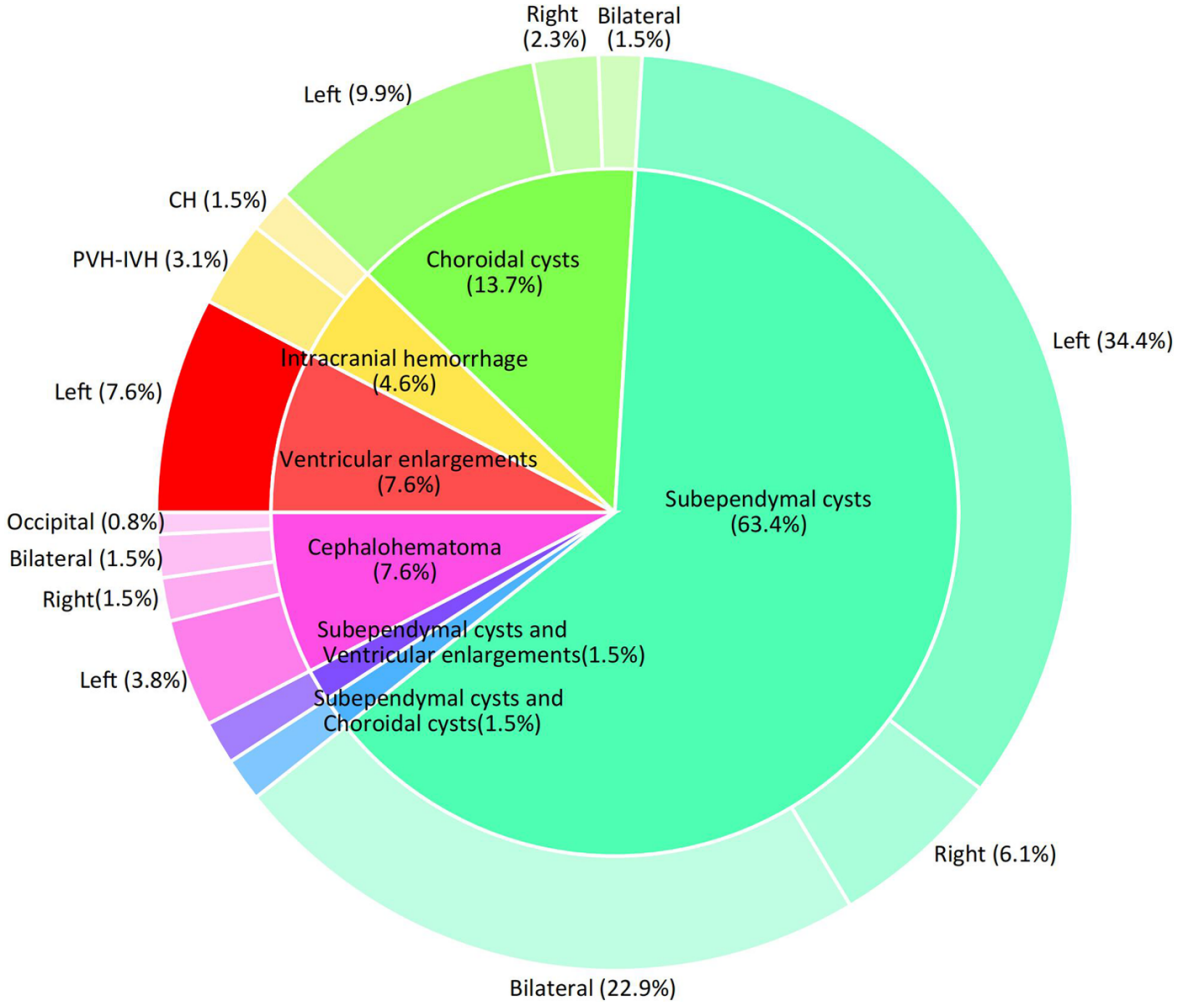
Disease distribution of CUS screening abnormalities. Note: Two cases of intraventricular hemorrhage were associated with subventricular cysts and one with a cephalohematoma.

Limited research has been conducted on the relationship between RDW-CV and the nervous system, particularly in neonates. Chugh et al. examined the correlation between RDW-CV and functional outcomes in subarachnoid hemorrhage in a cohort of 40 patients. Their findings indicated that higher RDW-CV levels were linked to unfavorable outcomes, suggesting that RDW may serve as a valuable prognostic indicator in patients (5). Increased RDW is associated with hematoma expansion in patients with intracerebral hemorrhage (14, 15). Another study found that non-survivors of intracerebral hemorrhage had higher RDW levels during the first week than survivors (16). In this study, we investigated the correlation between RDW-CV and CUAs in patients with neonatal hyperbilirubinemia with a gestational age of ≥35 weeks. Our findings showed that the risk of CUAs tended to increase with increasing RDW-CV levels, consistent with the literature. We controlled for potential confounders in our multivariable analysis, as detailed in Sub-Table 1, to isolate RDW-CV's effect on CUAs risk. Sensitivity analysis confirmed the robustness of RDW-CV's association with CUAs, independent of common confounders. Other noteworthy findings emerged from this study. First, subventricular cysts accounted for the largest number of CUAs. Second, the incidence of left-hemisphere abnormalities was significantly higher than that of the right and bilateral hemispheres (Figure 3). The exact reason for this is not clear, and further investigation is warranted to validate our findings and elucidate the underlying mechanisms.

However, Kelly et al. (17) reported that although RDW is a prognostic factor for survival in many inflammatory, prothrombotic, and neoplastic diseases, preoperative RDW was not associated with overall survival in patients with glioblastoma (GBM). These differences in findings may be related to differences in population heterogeneity, sample size, and diversity of unknown factors that could contribute to body development. The above studies suggest that although ultrasound screening is abnormal, further follow-up is needed to determine any complications.

Regarding potential mechanisms and clinical implications, emerging studies have suggested a link between RDW and inflammation. RDW has been shown to increase in inflammatory states (18, 19). Some studies have shown that specific inflammatory cytokines, including IL-6, ESR, and CRP, are positively correlated with RDW. A possible underlying mechanism is that pro-inflammatory cytokines suppress maturing RBCs, allowing newer and larger reticulocytes to enter the bloodstream, leading to increased RDW (20, 21).

Inflammation plays a vital role in neurological development. Many studies show the presence of an association between biomarkers of oxidative stress and brain damage in newborns (22, 23). Klein et al. (24) reported that systemic inflammation causes chronic activation of microglia and maldevelopment of the cerebellum in mice. Yanni et al. (25) noted that placental inflammation followed by postnatal systemic inflammation predisposes individuals to indicators of white matter damage on CUS, cerebral palsy, low developmental indices, and microcephaly. Therefore, inflammation may be a significant factor connecting RDW and traumatic brain injury. Further research is required to clarify the potential association between RDW and CUAs.

This study has several strengths. It investigated the correlation between RDW-CV and CUAs, an area that has not received substantial attention from clinicians. This analysis has the potential to enhance clinicians’ evaluation of neurological abnormalities in children with neonatal hyperbilirubinemia. Nonetheless, the present study has some limitations that warrant consideration. First, it is important to acknowledge that an observational study differs from a randomized controlled trial (RCT); thus, the findings obtained may diverge from the anticipated results of an RCT owing to inherent limitations in the study design. Therefore, our findings should be interpreted in the context of this distinction. Despite these limitations, the data obtained in our study offer valuable insights into the association between RDW-CV and CUAs, contributing additional evidence to this field of research.

## Conclusion

This study revealed a proportional relationship between a higher RDW-CV and an increased risk of CUAs in neonatal hyperbilirubinemia. These results demonstrate the importance of RDW-CV in neonatal hyperbilirubinemia. This study showed that RDW-CV may serve as a risk factor and predictor of CUAs. Further research is required to confirm this hypothesis.

## Data Availability

The raw data supporting the conclusions of this article will be made available by the authors, without undue reservation.
