# Supplementary material for: Association of red blood cell distribution width–coefficient of variation with cranial ultrasound abnormalities in neonatal hyperbilirubinemia: a retrospective cross-sectional study
Source: Front Pediatr. 2025 Jan 29;12:1488731. doi: 10.3389/fped.2024.1488731 (PMC11814458; doi:10.3389/fped.2024.1488731)
Supplement: Supplementary file 1 [file Table1.doc]

**Sub-Table1. Univariate analysis for CUAs in neonatal hyperbilirubinemia**

| Covariate | OR(95% CI) | P-value |
| --- | --- | --- |
| RDW-CV | 1.248 (1.042~1.494) | 0.0159 |
| Age, h | 0.999 (0.996~1.001) | 0.3178 |
| sex |  |  |
| Male | 1(Ref) |  |
| Female | 1.175 (0.788~1.752) | 0.4284 |
| Birth Weight, g | 1.000 (1.000~1.001) | 0.2924 |
| Week of Gestation, week | 0.823 (0.705~0.962) | 0.0142 |
| Delivery mode, n (%) |  |  |
| Vaginal delivery | 1(Ref) |  |
| C - section | 0.922 (0.603~1.410) | 0.7087 |
| Vaginal delivery to C - section | 1.673 (0.391~7.151) | 0.4875 |
| WBC, 10e9/L | 0.937 (0.876~1.003) | 0.0621 |
| HCT, % | 0.984 (0.952~1.017) | 0.3316 |
| PLT, 10e9/L | 0.998 (0.996~1.001) | 0.1905 |
| ALT, U/L | 0.992 (0.959~1.026) | 0.6385 |
| TBIL, umol/L | 0.998 (0.995~1.002) | 0.3771 |
